# Upregulated microRNA-429 confers endometrial stromal cell dysfunction by targeting HIF1AN and regulating the HIF1A/VEGF pathway

**DOI:** 10.1515/med-2023-0775

**Published:** 2023-10-13

**Authors:** Rong Zheng, Yulan Liu, Yan Lei, Yan Yue

**Affiliations:** Department of Gynecology, Maternal and Child Health Hospital of Hubei Province, Tongji Medical College, Huazhong University of Science and Technology, Wuhan 430070, Hubei, China; Department of Gynecology, Maternal and Child Health Hospital of Hubei Province, Tongji Medical College, Huazhong University of Science and Technology, No. 745 Wu Luo Road, Hongshan District, Wuhan 430070, Hubei, China

**Keywords:** endometriosis, endometrial stromal cells, miR-429, HIF1AN, HIF1A/VEGF pathway

## Abstract

Endometriosis (EM) is a prevalent estrogen-dependent disorder that adversely affects the life quality of many reproductive-age women. Previous evidence has suggested the significant role of miR-429 in EM; however, its molecular mechanisms underlying EM pathogenesis are unclarified. Human endometrial stromal cells (HESCs) were identified using immunofluorescence staining and flow cytometry. A mouse EM model was established by endometrial auto-transplantation. RNA and protein expression of molecules was examined using real-time quantitative polymerase chain reaction and western blotting, respectively. *In vitro* functional experiments showed that inhibiting miR-429 restrained HESC proliferation, migration, and invasiveness. Luciferase reporter assay confirmed that miR-429 targeted hypoxia-inducible factor 1 subunit alpha inhibitor (HIF1AN) in HESCs. HIF1AN silencing offset the negative regulation of miR-429 inhibition on the HIF1A/vascular endothelial growth factor (VEGF) signaling pathway. *In vivo* experiments showed that depletion of miR-429 attenuated ectopic lesion development in the mouse EM model. Collectively, suppressing miR-429 hinders the invasive behaviors of HESCs and EM progression in mice by targeting HIF1AN and regulating the HIF1A/VEGF signaling pathway.

## 1 Introduction

Endometriosis (EM) is a prevalent gynecological disorder referring to the extra-uterine growth of endometrial stromal or glandular tissue [1]. It affects approximately 10% of reproductive-age women and can cause symptoms including chronic pelvic pain, dysmenorrhea, dyspareunia, and infertility [2,3]. Although benign, EM shares very similar biological characteristics with malignant tumors and has been considered a precursor lesion of several malignancies, including ovarian cancer [4]. The pathogenesis of EM remains unclear. Accumulating evidence has indicated that the increased proliferation, invasion, migration, and adhesion of endometrial stromal cells (ESCs) contribute to the ectopic implantation of endometrial tissue and the progression of endometriotic lesions [5]. Repression of the invasive behaviors of ESCs mitigates EM development [6].

MicroRNAs (miRNAs) are small endogenous nonprotein-coding fragments with approximately 22 nucleotides and mediate gene expression post-transcriptionally [7]. Circulating miRNAs are considered promising diagnostic biomarkers for diseases largely owing to their high stability in human fluids [8]. As estimated, miRNAs control more than 50% of the mammalian protein-coding genes involved in various biological processes [9]. Accumulating evidence has suggested that targeting miRNAs is a promising therapeutic strategy for disease treatment [10]. Recent reports have suggested that miRNAs are implicated in the pathophysiology of EM. For instance, rhein exhibits an anti-proliferative role in EM by repressing miR-135 [11]. Kai et al. demonstrated that miR-210 is downregulated in ectopic endometrial samples and overexpressing miR-210 attenuates the proliferation and migration of ectopic endometriotic epithelial cells [12]. Moreover, previous evidence has indicated the upregulation of miR-429 in EM patients, and Berberine can impede the invasive phenotypes of ESCs by downregulating miR-429 [13,14]. These results indicate the significant role of miR-429 in EM. Nevertheless, the underlying molecular mechanisms of miR-429 in regulating EM progression are unclear.

Hypoxia-inducible factor 1 subunit alpha inhibitor (HIF1AN) gene is located at chromosome 10q24.31 and encodes an asparaginyl hydroxylase that regulates the activity of key biological modulators via hydroxylation [15]. HIF1AN is a well-known negative regulator of HIF1A [16]. It was demonstrated that HIF1A is upregulated in the ectopic endometrium of EM patients and contributes to ESC invasiveness and migration by enhancing autophagy [17]. Additionally, vascular endothelial growth factor (VEGF) is a downstream target of HIF1A. HIF1A binds to hypoxia-responsive elements in the promoter of VEGF, consequently leading to VEGF upregulation [18]. The VEGF level in the peritoneal fluid of EM patients is markedly increased in comparison to that of health controls [19]. Ye et al. demonstrated that miR-21-5p targets and downregulates HIF1AN, consequently leading to the activation of the HIF1A/VEGF pathway in choriocarcinoma [20]. Herein, we investigated whether miR-429 regulated the invasive behaviors of ESCs via the HIF1AN-mediated HIF1A/VEGF pathway.

Herein, we explored miR-429 functions and molecular mechanisms in regulating the invasive phenotypes of ESCs. It was hypothesized that miR-429 might mediate ESC phenotypes by regulating downstream molecules. Our results might help deepen the understanding of molecular mechanisms underlying EM pathogenesis.

## 2 Materials and methods

### 2.1 Cell culture and characterization

Human endometrial stromal cells (HESCs) purchased from Procell (Wuhan, China) were incubated in Dulbecco's modified Eagle's medium (Gibco, Carlsbad, NY, USA) containing 10% fetal bovine serum (FBS, Gibco) and 1% penicillin–streptomycin (Gibco) and maintained at 37°C in a humidified atmosphere with 5% CO_2_. Cells at passage 3 were used for subsequent experiments. Cell morphology was observed under a light microscope (Olympus, Tokyo, Japan). HESCs were identified by immunofluorescence (IF) staining for vimentin and cytokeratin 7 (CK7) as well as flow cytometry using antibodies against CD29, CD73, CD45, and CD31 (all from Abcam, Shanghai, China).

### 2.2 Cell transfection

For knockdown assays, miR-429 inhibitor or its negative control NC inhibitor, short hairpin RNA targeting HIF1AN (sh-HIF1AN) or control sh-NC purchased from GenePharma (Shanghai, China) were transfected into HESCs using Lipofectamine 3000 (Invitrogen, Carlsbad, CA, USA). Cells were harvested for further analysis 48 h post-transfection.

### 2.3 Quantitative reverse transcription PCR (RT-qPCR)

Total RNA isolation from HESCs or endometriotic lesions was performed using TRIzol reagent (Invitrogen). RNA samples were reverse-transcribed using iScript cDNA Synthesis Kit (Bio-Rad, Hercules, CA, USA) to produce cDNA. RT-qPCR was implemented using SYBR Green Master (Roche, Mannheim, Germany). Normalized to U6 and GAPDH, miR-429 and mRNA expression was quantified with the 2^−ΔΔ*C*t^ method. Primer sequences are shown in Table 1.

**Table 1:** Primers used for RT-qPCR

Gene	Sequence (5′–3′)	
miR-429	Forward	AGGTCT CTGAGGGTCAAGCA
	Reverse	CTGGTTGAAAAGCATGAGCA
TOB1	Forward	TGCTACCTGAACAAGATCAC
	Reverse	ACTTGGATTTCAAGCTGCA
RTN4	Forward	CCATTCAGGGCATATCTGGA
	Reverse	ATGACCAAGAGCAGAATTACTG
DNAJB9	Forward	CATGAAGTACCACCCTGAC
	Reverse	GAGTGTTTCATATGCTTCTGC
HYOU1	Forward	AGTGCCCATGGAAATTGTC
	Reverse	CTTAATCGCCATGCTTGCT
XIAP	Forward	GGAGGGCTAACTGATTGGA
	Reverse	TAACAGATATTTGCACCCTGGA
HIF1AN	Forward	TTAAGCCGAGGTCCAACAG
	Reverse	TGCTGCAGATACAACCTCTC
SYNJ1	Forward	GCCTCATAGTGGAAACTAGG
	Reverse	TTTCTGCAGATGAGAGCAC
U6	Forward	ATACAGAGAAAGTTAGCACGG
	Reverse	GGAATGCTTCAAAGAGTTGTG
GAPDH	Forward	TCAAGATCATCAGCAATGCC
	Reverse	CGATACCAAAGTTGTCATGGA

### 2.4 Cell counting kit-8 (CCK-8) assay

Transfected HESCs (1 × 10^5^ cells/well) were inoculated into 96-well plates for 24, 48, and 72 h. Afterward, 10 μL of CCK-8 solution was added to each well (Solarbio, Beijing, China) and incubated for another 2 h. The 450 nm absorbance was examined using a microplate reader (Thermo Scientific, Waltham, MA, USA).

### 2.5 EdU assay

Transfected HESCs in 96-well plates were incubated with EdU (5-ethynyl-2′-deoxyuridine) solution (50 μM; Beyotime, Shanghai, China) for 2 h. After fixing with 4% paraformaldehyde, cells were stained with Apollo solution and DAPI solution (4',6-diamidino-2-phenylindole; Solarbio) for 30 min. The stained cells were observed under a fluorescence microscope (Olympus).

### 2.6 Scratch assay

Transfected HESCs were plated in six-well plates and then the confluent monolayer cells were scratched using a 10 μL Eppendorf tip. The plates were washed twice with phosphate buffer saline for removing exfoliated cells. After 24 h, wound closure was examined using a microscope (Olympus) and analyzed with ImageJ software (NIH, Bethesda, MD, USA).

### 2.7 Transwell assay

HESCs in serum-free medium were placed into the upper Matrigel-covered Transwell chamber (24 wells, 8 μm pore size, Corning, NY, USA), with medium containing 10% FBS added to the lower chamber. After culturing for 24 h, the invaded cells in the lower chamber were fixed with 10% formaldehyde, dyed with 0.1% crystal violet, and counted under an inverted microscope (Olympus).

### 2.8 Western blotting

Protein extraction from HESCs or endometriotic lesions was carried out using RIPA lysis buffer (Solarbio), and a bicinchoninic acid assay kit (Solarbio) was utilized for measuring the protein concentration. Protein samples (20 μg) were dissolved with 10% sodium dodecyl sulfate-polyacrylamide gel electrophoresis, blotted onto polyvinylidene fluoride membranes (Beyotime), and blocked with 5% defatted milk. Next, the membranes were incubated overnight with anti-HIF1AN (ab92498, 1:5,000), anti-HIF1A (ab179483, 1:1,000), anti-VEGF (ab214424, 1:1,000), and anti-GAPDH (ab22555, 1:1,000) primary antibodies (all from Abcam) at 4℃, and then incubated at room temperature with the secondary antibody (ab6721, 1:2,000, Abcam) for 2 h. Protein bands were visualized with the enhanced chemiluminescence detection kit (Solarbio) and quantified with ImageJ software (NIH).

### 2.9 Luciferase reporter assay

The ENCORI database shows the putative binding site between miR-429 and HIF1AN. HIF1AN wild type (Wt) or mutant (Mut) 3′-UTR sequence containing miR-429 binding site was subcloned into pmirGLO vector (Promega, Madison, WI, USA). HESCs were co-transfected with these vectors and miR-429 or NC inhibitor using Lipofectamine 3000 (Invitrogen). A dual luciferase reporter assay system (Promega) was utilized for luciferase activity measurement 48 h post-transfection.

### 2.10 Mice

Female BALB/c nude mice (6 and 7 weeks, 18–22 g) were purchased from Cavens Laboratory Animal Co., Ltd (Changzhou, China) and housed in a specific pathogen-free environment (temperature, 23 ± 2℃, humidity, 50–60%, 12 h light/dark cycle) with free access to food and water. The mice were allowed to acclimatize for 1 week before experiments. All animal experiments were conducted following the NIH Guidelines for the Care and Use of Laboratory Animals and approved by the Ethics Committee of Wuhan Myhalic Biotechnology Co., Ltd (202211005).

### 2.11 Mouse EM model

The mouse EM model was established by endometrial auto-transplantation according to previous description [21]. Briefly, all mice were anesthetized by intraperitoneal injection of 1% pentobarbital sodium (40 mg/kg). Then, a small midline incision was made on the abdomen, and the left uterine horn was excised and immediately placed in a physiological saline solution. The endometrium was carefully separated from muscles and cut into two segments (5 mm × 5 mm). A subcutaneous pocket was created on each side of the abdominal wall and the uterine segments were placed in the spaces with the endometrium facing the abdominal muscle. Then, the abdominal incision was sutured. One day after the surgery, the mice were randomly divided into two groups (*n* = 6/group). The mice were injected intraperitoneally with 90 μg of NC inhibitor or miR-429 inhibitor and 20 μL of *in vivo*‐jet PEI delivery reagent (Polyplus Transfection, Illkirch, France) in 5% glucose every 2 days, respectively. After 2 weeks, the mice were euthanized under anesthesia, and endometriotic lesions were collected for subsequent experiments. All results were assessed by two investigators blinded to the group treatments.

### 2.12 Measurement of estradiol levels

The level of estrogen estradiol in the homogenates of endometriotic lesions was estimated using an enzyme-linked immunosorbent assay (ELISA) kit (Beyotime) following the manufacturer’s instructions.

### 2.13 Statistical analysis

All experiments were repeated at least three times. Data are presented as mean ± standard deviation. Student’s *t*-test or one-way ANOVA was carried out for evaluating statistical differences among groups using SPSS 25.0 software (IBM, Armonk, MA, USA). *p* < 0.05 was defined as statistically significant.

## 3 Results

### 3.1 Identification of HESCs

**Figure 1:**
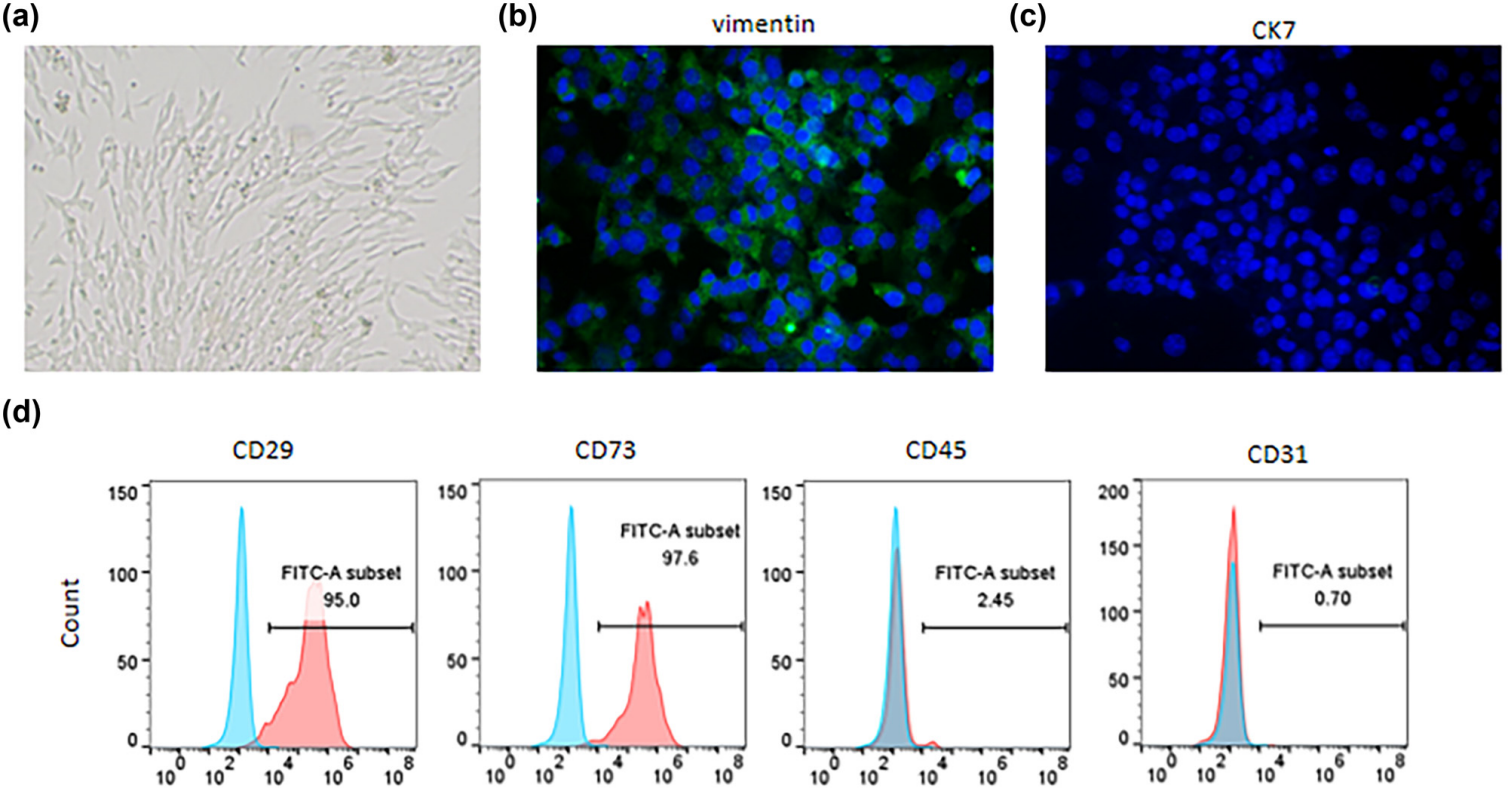
Identification of HESCs. (a) Morphology of HESCs under a light microscope. (b) and (c) Representative images of IF staining for examining vimentin (b) and CK7 (c) expression in HESCs. (d) Flow cytometry for detecting cell surface markers of HESCs.

As shown in Figure 1a, HESCs exhibited a fibroblast-like morphology. IF staining displayed positive staining of vimentin, a stromal cytoskeletal marker, and negative staining of CK7, an epithelial marker (Figure 1b and c). Additionally, HESCs were positive (≥95%) for the mesenchymal cell markers CD29 and CD73 and negative (<5%) for the hematopoietic cell markers CD45 and CD31, as indicated by flow cytometry (Figure 1d). The above results confirmed the stromal cell characteristics of HESCs.

### 3.2 miR-429 effect on the HESC growth

**Figure 2:**
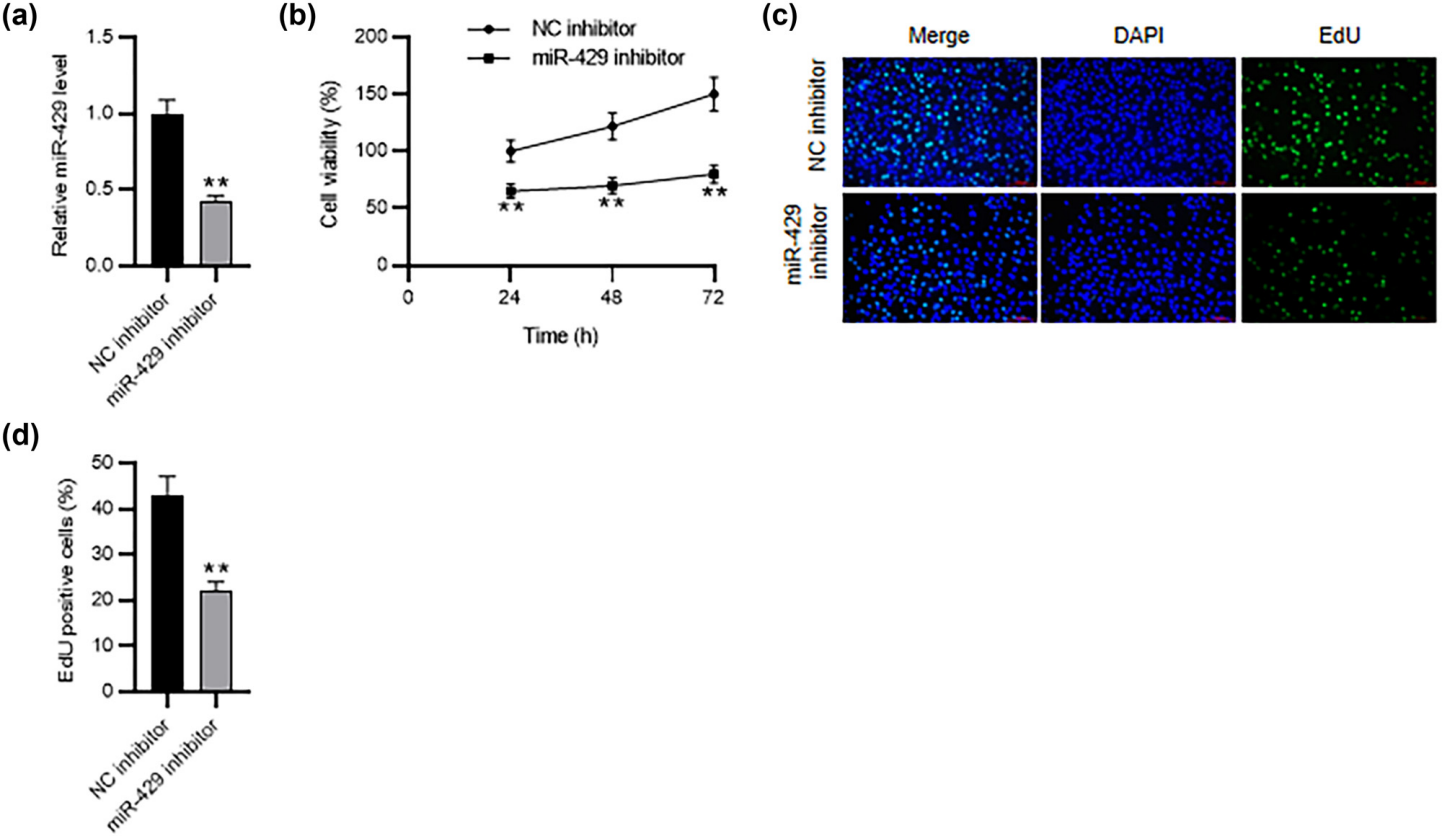
Knocking down miR-429 represses HESC growth. HESCs were transfected with miR-429 or NC inhibitor. (a) RT-qPCR for analyzing miR-429 knockdown efficiency. (b) CCK-8 assay for determining HESC viability. (c) and (d) EdU assay for evaluating HESC proliferation. **p < 0.01.

To identify the effect of miR-429 on the phenotypes of HESCs, we knocked down miR-429 in HESCs. Notably, miR-429 expression was reduced in miR-429 inhibitor-treated HESCs compared to that in NC inhibitor-treated HESCs, confirming the successful transfection of miR-429 inhibitor (Figure 2a). The growth of HESCs was examined with CCK-8 and EdU assays. As displayed by the results, the proliferative capability of HESCs was markedly repressed after inhibiting miR-429 (Figure 2b–d).

### 3.3 Effect of miR-429 on HESC migration and invasiveness

**Figure 3:**
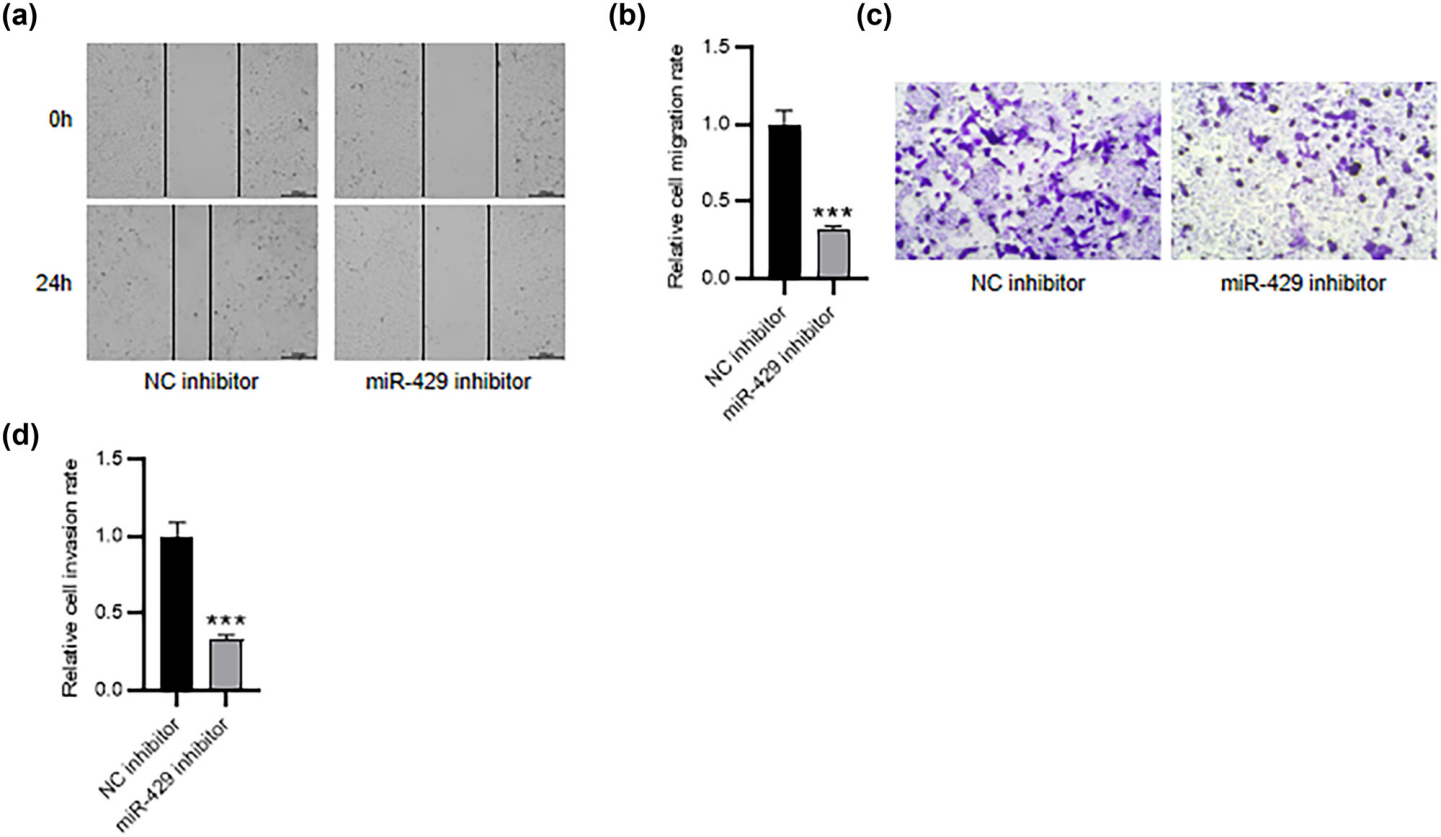
Inhibiting miR-429 restrains HESC migration and invasiveness. (a) and (b) The scratch assay for examining HESC migratory capability. (c) and (d) Transwell assay for detecting HESC invasiveness. ***p < 0.001.

The effect of miR-429 on HESC migration and invasiveness was also investigated. The scratch assay revealed that miR-429 depletion prominently restrained the migratory ability of HESCs (Figure 3a and b). In parallel, HESC invasiveness was significantly impeded by miR-429 inhibitor, as suggested by Transwell assay (Figure 3c and d). Collectively, inhibiting miR-429 restrained HESC migration and invasiveness.

### 3.4 miR-429 targets HIF1AN in HESCs

**Figure 4:**
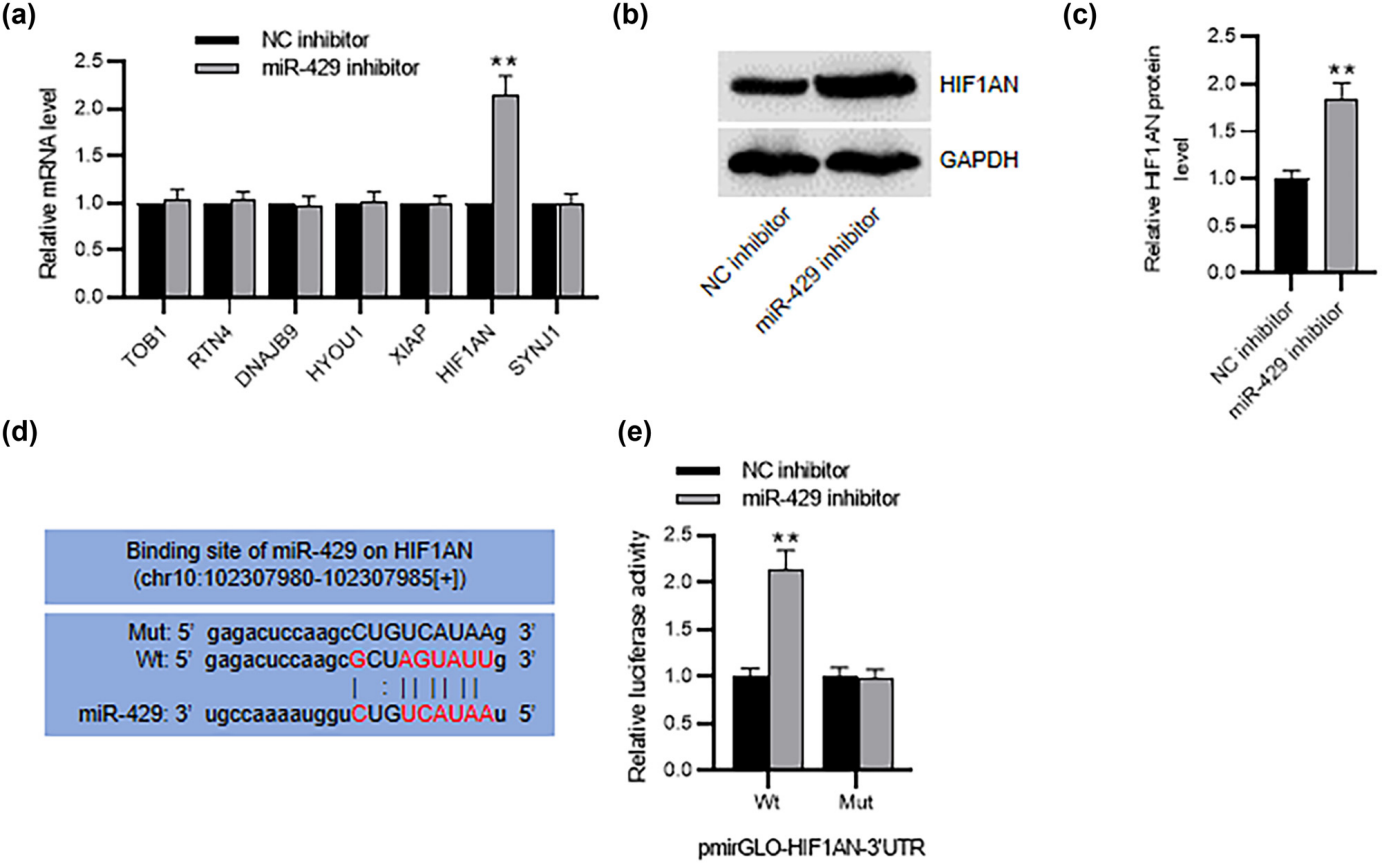
miR-429 targets HIF1AN in HESCs. (a) RT-qPCR for evaluating mRNA levels of miR-429 candidate targets. (b) and (c) Western blotting of HIIF1AN protein expression in miR-429-silenced HESCs. (d) The predicted binding site of miR-429 on HIF1AN by ENCORI. (e) Luciferase reporter assay for elucidating the interaction between miR-429 and HIF1AN in HESCs. **p < 0.01.

To explore the potential molecular mechanism of miR-429 in HESCs, we explored its downstream genes utilizing the ENCORI database. With the screening condition of the number of supported AGO CLIP-seq experiments (AgoExpNum) > 35, seven candidate genes were screened out. RT-qPCR analysis demonstrated that among the seven genes, only HIF1AN was significantly upregulated in miR-429-inhibited HESCs (Figure 4a). Hence, HIF1AN was selected for further analysis. Suppression of miR-429 markedly upregulated HIF1AN protein expression in HESCs, as indicated by western blotting (Figure 4b and c). The ENCORI database predicts the binding site of miR-429 on HIF1AN (Figure 4d). We then mutated the predicted binding site on HIF1AN and performed the luciferase reporter assay. Notably, the luciferase activity of HIF1AN 3′-UTR was markedly elevated in miR-429-depleted HESCs but was almost unchanged after mutation (Figure 4e), confirming the interaction between HIF1AN and miR-429.

### 3.5 miR-429/HIF1AN axis regulates the HIF1A/VEGF signaling pathway

**Figure 5:**
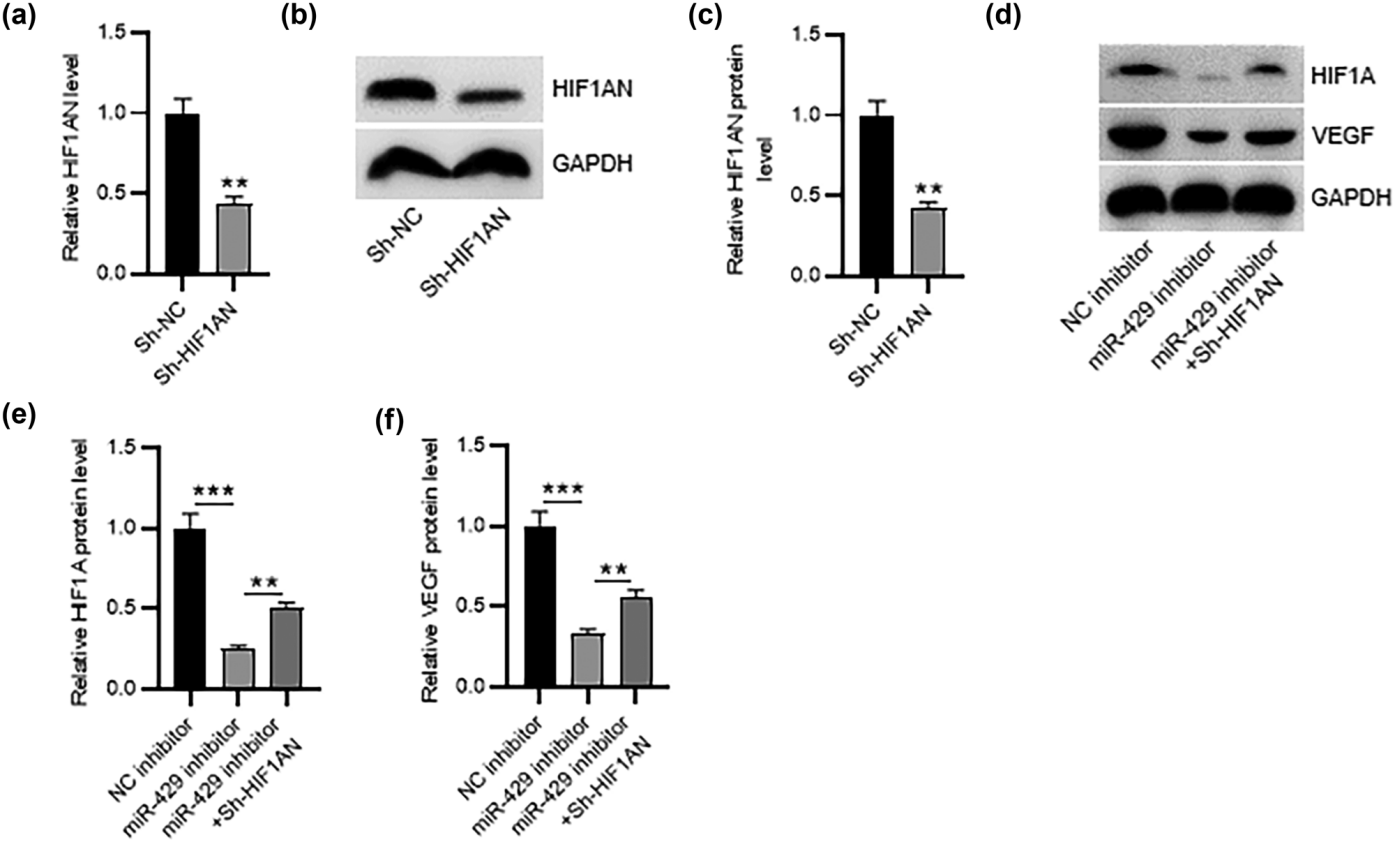
The miR-429/HIF1AN axis regulates the HIF1A/VEGF signaling pathway. (a)–(c) RT-qPCR and western blotting for determining HIF1AN silencing efficiency. (d)–(f) Western blotting for measuring HIF1A and VEGF protein expression in HESCs with indicated treatments. **p < 0.01 and ***p < 0.001.

As displayed in Figure 5a–c, HIF1AN mRNA and protein expression levels were prominently reduced in HESCs with transfection of sh-HIF1AN. Then, we explored the effect of the miR-429/HIF1AN axis on the HIF1A/VEGF signaling pathway using western blotting. Notably, miR-429 inhibition induced the downregulation of HIF1A and VEGF in HESCs, whereas these effects were counteracted by HIF1AN silencing (Figure 5d–f), indicating that the miR-429/HIF1AN axis might contribute to HIF1A/VEGF signaling.

### 3.6 Effect of miR-429 on EM progression *in vivo*

**Figure 6:**
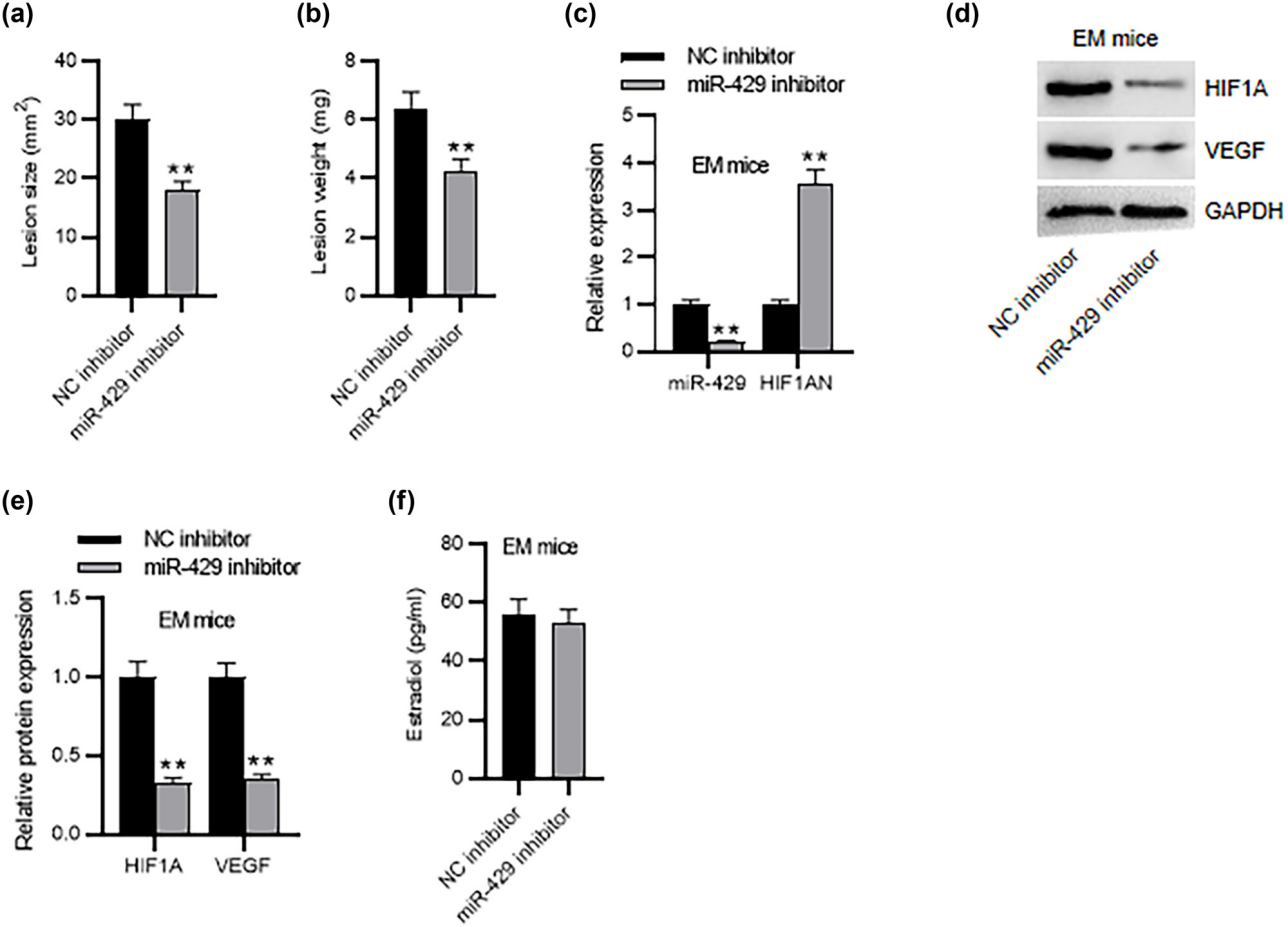
Inhibition of miR-429 impedes EM progression *in vivo*. Evaluation of the (a) size and (b) weight of EM lesions. (c) RT-qPCR analysis of miR-429 and HIF1AN expression in endometriotic tissues of EM mice. (d) and (e) Western blotting for assessing HIF1A and VEGF protein levels in endometriotic tissues of EM mice. (f) Assessment of estradiol levels in mouse endometriotic tissues by ELISA. *n* = 6 mice per group. **p < 0.01.

To further verify the effect of miR-429 on EM progression, a mouse EM model was established by endometrial auto-transplantation. The endometriotic lesions were collected from the two groups. As shown by the results, the ectopic lesions in the miR-429 inhibitor-treated group were significantly smaller in size and lighter in weight as compared to those in the NC inhibitor-treated group (Figure 6a and b), indicating that inhibition of miR-429 impeded EM development in mice. Moreover, in comparison to mice treated with NC inhibitor, miR-429 expression was markedly downregulated while the HIF1AN expression level was increased in the endometriotic tissues of mice treated with miR-429 inhibitor (Figure 6c), confirming that miR-429 negatively regulated HIF1AN in EM mice. Consistent with the *in vitro* results, animal experiments displayed that depletion of miR-429 reduced HIF1A and VEGF protein levels in the endometriotic tissues of EM mice (Figure 6d and e). Additionally, we estimated whether miR-429 impacted the estradiol level in EM mice. The results showed that there was no significant difference in the estradiol level between the two groups (Figure 6f). These data demonstrated that inhibiting miR-429 could attenuate EM progression in the mouse model.

## 4 Discussion

EM is an estrogen-dependent disorder that is similar to malignant tumors in biological behaviors, such as excessive proliferation, metastasis, and recurrence [22]. Despite decades of research into the pathogenesis of EM, the exact etiology of this disease is still incompletely understood [23]. Growing evidence has illustrated that non-coding RNAs, including miRNAs, act as critical regulators in the onset and progression of EM [24]. Many studies have illustrated that miR-429 participates in multiple biological processes and modulates disease progression. For instance, downregulated miR-429 in plasma exosomes mediates the PTEN/PI3K/Akt signaling pathway to suppress neuronal apoptosis after spinal cord injury [25]. Nguyen et al. proposed that miR-429 interacts with CFL2 and induces its downregulation, thus impairing myoblast differentiation [26]. Overexpressed miR-429 impedes glioblastoma cell growth and migration and promotes cell apoptosis by downregulating EGFR, BCL2, and MYC, which are several oncogenes of the ERBB pathway [27]. Importantly, it has been proposed that miR-429 expression is elevated in tissues of EM patients, and overexpressing miR-429 can reverse berberine-triggered suppression of the invasive phenotypes of HESCs [14]. Nonetheless, the underlying molecular mechanisms of miR-429 in regulating HESC phenotypes are unclarified. Consistent with the aforementioned evidence, our results revealed that the depletion of miR-429 prominently restrained the proliferation, invasiveness, and migration of HESCs. *In vivo* experiments further demonstrated that inhibiting miR-429 ameliorated ectopic lesion development in EM mice, indicating that miR-429 might exert a promoting effect on EM progression. Additionally, we found that inhibiting miR-429 expression did not significantly affect the estradiol level in the endometriotic tissues of mice, suggesting that miR-429 might affect EM progression not by directly regulating estrogen levels but by influencing invasive phenotypes of ESCs.

Mounting evidence has documented that miRNAs can interact with the 3′-UTRs of downstream targets via base pairing, thereby inducing reduced gene/protein expression [28]. As mentioned above, miR-429 participates in regulating the development of multiple human disorders by modulating downstream targets. Herein, we explored miR-429 target genes in HESCs using bioinformatics analysis and confirmatory experiments. As a result, HIF1AN was verified to be a target of miR-425. Multiple studies have illuminated the critical roles of HIF1AN in various pathophysiological events. Zhang et al. indicated that miR-212 facilitates renal interstitial fibrosis by repressing HIF1AN expression [29]. Upregulated HIF1AN exhibits a suppressive effect on osteoblast differentiation and calcification [15]. Knocking down HIF1AN enhances the proliferative and migratory potentials of human keloid fibroblasts [30]. However, whether it is implicated in EM has not been investigated. This study revealed that HIF1AN was upregulated in miR-429-depleted HESCs and endometriotic tissues of mice.

Moreover, previous reports have suggested that HIF1AN negatively regulates the HIF1A/VEGF signaling pathway and both HIF1A and VEGF have been indicated to be upregulated in EM patients [20,31]. HIFIA enhances the invasion and migration of HESCs by activating autophagy in EM [17]. These results indicate the significant role of HIF1AN in EM. Our results depicted that inhibiting miR-429 downregulated HIF1A and VEGF protein expression in HESCs and knocking down HIF1AN could offset this effect, suggesting that the miR-429/HIF1AN axis might promote the HIF1A/VEGF pathway in HESCs, thereby contributing to EM development.

In conclusion, we explored the miR-429 molecular mechanism in EM. Our results suggest that miR-429 facilitates HESC proliferation, migration, and invasiveness in EM by modulating the HIF1AN-mediated HIF1A/VEGF signaling pathway. These findings might deepen the understanding of the mechanism underlying EM pathogenesis. Additionally, further investigations are needed to clarify the role of the miR-429/HIF1AN axis in EM in the future.
